# Reduced Expression of Transcription Factor *AP-2α* Is Associated with Gastric Adenocarcinoma Prognosis

**DOI:** 10.1371/journal.pone.0024897

**Published:** 2011-09-26

**Authors:** Wei Wang, Lin Lv, Ke Pan, Yu Zhang, Jing-jing Zhao, Ju-gao Chen, Yi-bing Chen, Yong-qiang Li, Qi-jin Wang, Jia He, Shi-ping Chen, Zhi-wei Zhou, Jian-chuan Xia

**Affiliations:** 1 State Key Laboratory of Oncology in South China and Department of Experimental Research, Cancer Center, Sun Yat-sen University, Guangzhou, People's Republic of China; 2 Department of Gastric and Pancreatic Surgery, Cancer Center, Sun Yat-sen University, Guangzhou, People's Republic of China; 3 Department of Pathology, Cancer Center, Sun Yat-sen University, Guangzhou, People's Republic of China; Veterans Affairs Medical Center (111D), United States of America

## Abstract

**Background:**

This study aims to investigate the expression and prognostic significance of *activator protein 2α (AP-2α)* in gastric adenocarcinoma.

**Methodology/Principal Findings:**

*AP-2α* expression was analyzed using real-time quantitative PCR (RT-qPCR), western blotting, and immunohistochemical staining methods on tissue samples from a consecutive series of 481 gastric adenocarcinoma patients who underwent resections between 2003 and 2006. The relationship between *AP-2α* expression, clinicopathological factors, and patient survival was investigated. RT- qPCR results showed that the expression of *AP-2α* mRNA was reduced in tumor tissue samples, compared with expression in matched adjacent non-tumor tissue samples (P = 0.009); this finding was confirmed by western blotting analysis (P = 0.012). Immunohistochemical staining data indicated that *AP-2α* expression was significantly decreased in 196 of 481 (40.7%) gastric adenocarcinoma cases; reduced *AP-2α* expression was also observed in patients with poorly differentiated tumors (P = 0.001) and total gastric carcinomas (P = 0.002), as well as in patients who underwent palliative tumor resection (P = 0.004). Additionally, reduced expression of *AP-2α* was more commonly observed in tumors that were staged as T4a/b (P = 0.018), N3 (P = 0.006), and M1 (P = 0.008). Kaplan-Meier survival curves revealed that reduced expression of *AP-2α* was associated with poor prognosis in gastric adenocarcinoma patients (P<0.001). Multivariate Cox analysis identified *AP-2α* expression as an independent prognostic factor for overall survival (HR = 1.512, 95% CI = 1.127–2.029, P = 0.006).

**Conclusions/Significance:**

Our data suggest that *AP-2α* plays an important role in tumor progression and that reduced *AP-2α* expression independently predicts an unfavorable prognosis in gastric adenocarcinoma patients.

## Introduction

Gastric cancer is the fourth most common malignant tumor worldwide, with an estimated one million new cases every year [1]. More new cases of gastric cancer are diagnosed in China each year than in any other country [2]. Although current practice includes incorporating chemotherapy or radiation into surgical resection treatment protocols, gastric cancer survival rates remain poor [3]. Gastric cancer is a heterogeneous disease in both histology and genetics; hence, patient outcome is difficult to predict using classic histological classifications. Gastric carcinogenesis is considered to be a multifactorial and multistep process that involves the activation of oncogenes and the inactivation of tumor suppressor genes at different stages of gastric cancer progression. Recently, several new oncogenes and tumor suppressor genes associated with gastric cancer have been identified, which may be helpful for early diagnosis and for the development of targeted therapies [4], [5]. To improve patient prognosis, further understanding of the molecular mechanisms of cancer progression and the development of new therapeutic tools based on these mechanisms is required [4], [6], [7], [8], [9].

Transcription factors that have been implicated in the pathogenesis of malignancy could serve as novel therapeutic targets [10]. The activator protein-2 (AP-2) family of transcription factors comprises five 52-kDa isoforms (AP-2α, AP-2β, AP-2γ, AP-2δ, and AP-2ε) that are encoded by independent genes; AP-2α, AP-2β and AP-2γ are the most studied [11], [12], [13]. The isoforms share a common structure: a proline/glutamine-rich transactivation domain in the N-terminal region and a helix-span-helix domain in the C-terminal region, which mediates dimerization and site-specific DNA binding. Depending on the cellular context, the AP-2 transcription factors are individually associated either with cell differentiation and development or with cancer progression/regression. For example, loss of AP-2 expression results in the transition of melanoma cells to the metastatic phenotype, which indicates that AP-2 may have a tumor-suppressive role [14]. In addition, loss of AP-2 expression seems to be associated with malignant transformation and tumor progression and is independently associated with an elevated risk of subsequent metastatic behavior of stage I cutaneous malignant melanoma [10]. Furthermore, reduced nuclear AP-2 expression was shown to be associated with disease progression and increased metastatic capability in breast cancer. In addition, reduced nuclear AP-2 expression independently predicted an elevated risk of recurrent breast cancer [12]. In addition, reduced, or loss of, *AP-2a* expression has been reported in human cancers of breast, ovary, colon, brain, and prostate [13], [15], [16], [17], [18].

However, to the best of our knowledge, no previous reports exist concerning the expression status of *AP-2α* in primary gastric cancer, and the prognostic value of this protein in gastric cancer has not yet been assessed. Furthermore, it is important to investigate whether the correlation found in established cell lines grown in vitro can also be observed in clinical gastric cancer specimens.

In the present study, the expression of *AP-2α* in primary gastric adenocarcinoma was investigated using quantitative real-time PCR (RT-qPCR), western blotting and immunohistochemistry. The relationship between *AP-2α* expression and the clinicopathological factors, as well as the potential prognostic value of *AP-2α* expression in gastric cancer patients, were evaluated.

## Results

### *AP-2α* mRNA expression analyzed with RT-qPCR

**Figure 1:**
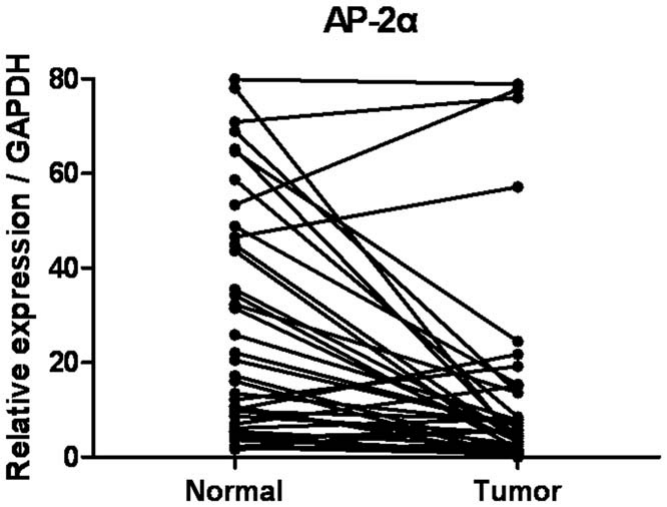
RT-qPCR analysis of *AP-2α* expression in gastric cancer patients. Relative expression of *AP-2α* in gastric cancer tumor tissues compared to adjacent non-tumor tissues (n = 41) assessed by RT-qPCR (P = 0.009).

The transcriptional levels of *AP-2α* were determined with RT-qPCR assays on 41 pairs of resected specimens (tumor tissue samples and matched adjacent non-tumor tissue samples) from gastric cancer patients. The *AP-2α* mRNA levels were significantly reduced in 30 (73%) tumor tissue samples, compared with levels in the adjacent non-tumor tissue samples (P = 0.009, Figure 1).

### *AP-2α* expression analyzed by Western blotting

**Figure 2:**
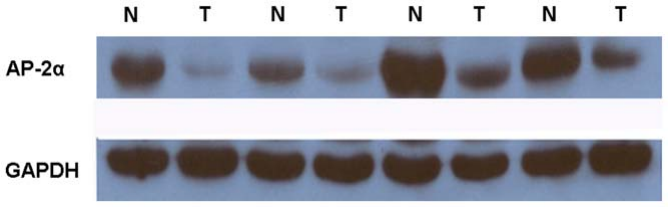
Western blotting analysis of *AP-2α* expression in gastric cancer patients. Relative expression of *AP-2α* in gastric cancer tumor (T) tissues compared to adjacent non-tumor (N) tissues (n = 41) assessed by western blotting, (P = 0.012).

The *AP-2α* protein levels in the resected gastric cancer samples were determined with Western blotting. As shown in Figure 2, a decrease in *AP-2α* expression was detected in 28 (68%) of the 41 tumor tissue samples, compared with expression in the matched adjacent non-tumor tissue samples (P = 0.012). These findings were consistent with those of the RT-qPCR.

### The association between *AP-2α* expression, based on immunohistochemical staining, and clinicopathological characteristics

To gain further insight into the effect and prognostic value of *AP-2α* expression in gastric cancer patients, paraffin-embedded tissue sections (n = 481) with histopathologically confirmed gastric adenocarcinoma were examined using immunohistochemistry. The levels of *AP-2α* immunoreactivity varied between the tumor tissue samples and the adjacent non-tumor tissue samples. Positive *AP-2α* expression was localized to the cytoplasm in 285 (59.3%) of the resected tumor tissue samples, whereas the remaining 196 cases (40.7%) displayed reduced cytoplasmic *AP-2α* expression (Table 1).

**Table 1:** Relationship between AP-2α expression and clinicopathologic features of patients with gastric cancer.

Variables	Number	AP-2α expression		*P* value
		Low	High	
**Age(years)**				0.192
≤60	270	117	153	
>60	211	79	132	
**Gender**				0.446
Male	326	129	197	
Female	155	67	88	
**Tumor size (cm)**				0.449
≤5.0	309	122	187	
>5.0	172	74	98	
**Histological grade**				0.001*
Well differentiated (G1)	18	3	15	
Moderately differentiated (G2)	166	45	121	
Poorly differentiated (G3)	297	148	149	
**Location**				0.002*
Proximal	271	106	165	
Distant	186	72	114	
Total	24	18	6	
**Radical resection**				0.004*
Yes	410	156	254	
No	71	40	31	
**Tumor invasion (T)**				0.018*
T1	35	12	23	
T2	43	13	30	
T3	98	33	65	
T4a	256	109	147	
T4b	49	29	20	
**Nodal status (N)**				0.006*
N0	135	44	91	
N1	94	36	58	
N2	107	40	67	
N3	145	76	69	
**Metastasis status (M)**				0.008*
M0	425	164	261	
M1	56	32	24	
**TNM Staging**				<0.001*
Stage I	50	14	36	
Stage II	118	35	83	
Stage III	256	114	142	
Stage IV	57	33	24	

*Statistically significant (*P*<0.05).

**Figure 3:**
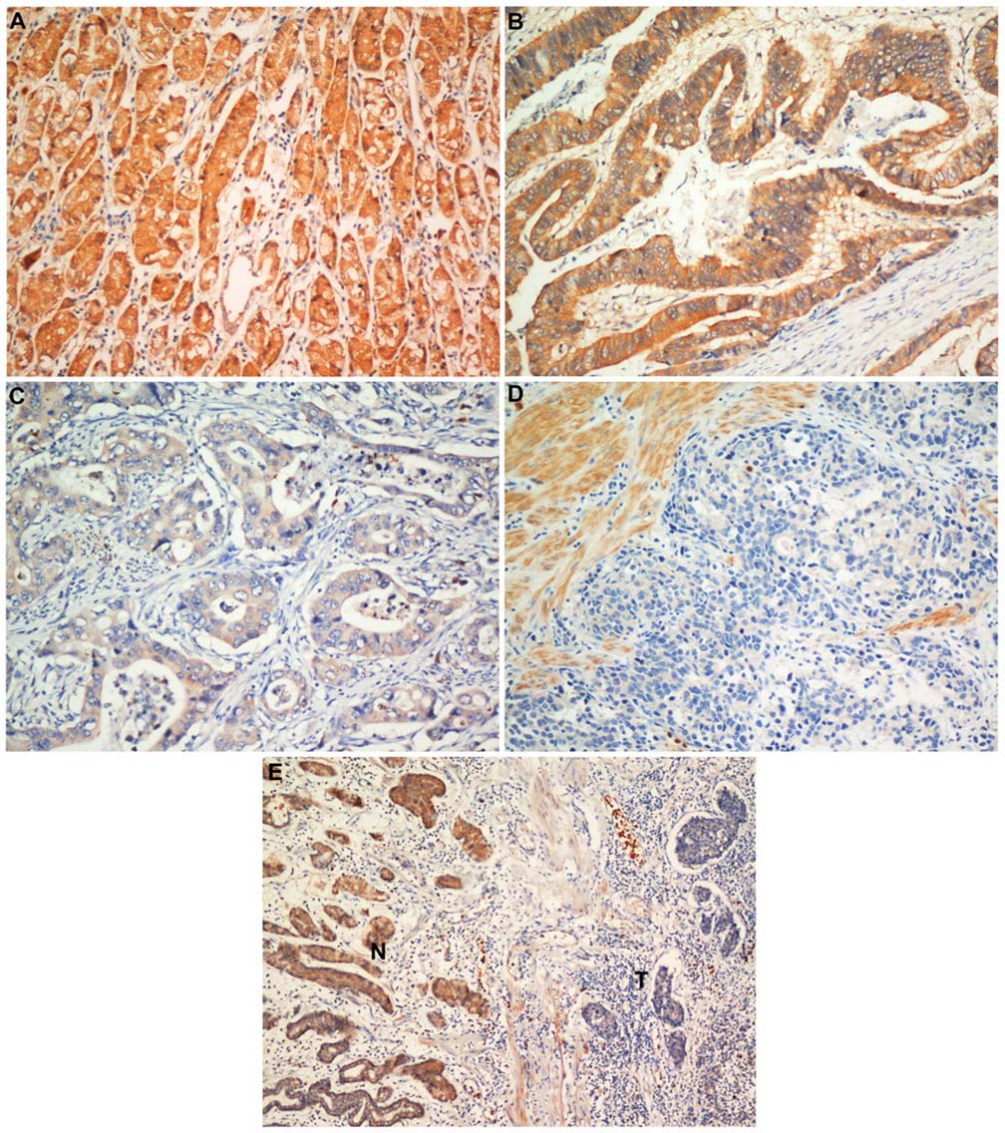
Immunohistochemical detection of the *AP-2α* protein expression in gastric cancer and surrounding non-tumor tissues. (A) Normal gastric tissues, scored as *AP-2α* (+++); (B) Well-differentiated gastric cancer, scored as *AP-2α* (++) according to the criteria defined in material and methods section; (C) moderately differentiated gastric cancer, scored as *AP-2α* (+); (D) poorly differentiated gastric cancer, scored as *AP-2α* (−); (E) Immunostaining of gastric cancer and adjacent non-tumor tissues showing a sharp contrast between infiltrative tumor areas of negative staining and the adjacent tissue of positive staining. Original magnification: A–D×200; E×100.

Based on our categories defined in the aforementioned methods, *AP-2α* expression was significantly reduced in patients with poorly differentiated carcinoma (G3) (P = 0.001) or with total gastric carcinoma (P = 0.002) and in the patients who underwent palliative tumor resection (P = 0.004). Reduced *AP-2α* expression was also observed significantly more frequently in tumors with deeper invasion (T4a and T4b) (P = 0.018) and in cases with distant metastases (M) (P = 0.008) and increased numbers of lymph node metastases (N3) (P = 0.006). Furthermore, 114 of 256 (44.5%) patients with stage III disease and 33 of 57 (57.9%) patients with stage IV disease had low *AP-2α* expression (P<0.001). The representative photomicrographs are shown in Figure 3.

### Correlation between *AP-2α* expression based on immunohistochemistry and patient survival

**Figure 4:**
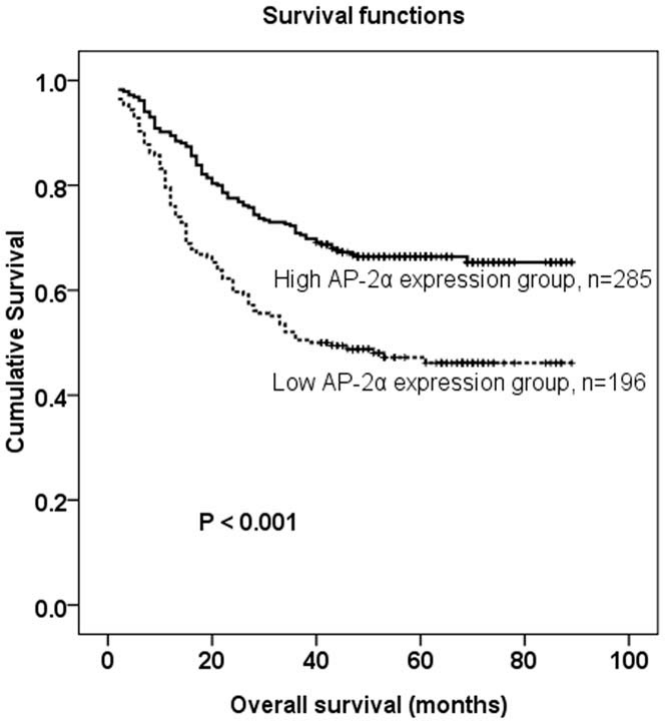
Kaplan–Meier survival curves of gastric cancer patients (n = 481) after surgical resection. Decreased *AP-2α* expression correlated with poor patient survival. Patients in high *AP-2α* expression group exhibited significantly better survival than the low *AP-2α* expression group (log-rank test: *P*<0.001).

The median survival time of the 481 gastric cancer patients was 46 months (range 3–89 months). The overall survival rate in the high *AP-2α* expression group were significantly improved compared to the low expression group (65.3 vs. 46.1%, P<0.001, Figure 4).

### Univariate and multivariate analyses

Univariate and multivariate analyses were performed to compare the impact of *AP-2α* expression and other clinicopathological parameters on prognosis. Based on univariate analysis that included all 481 patients, 9 factors were found to have statistically significant associations with overall survival; these included tumor location, tumor size, histological grade, radical resection, *AP-2α* expression, chemotherapy, and the T, N, and M stage based on the 7^th^ edition of the UICC TNM classification (Table 1). All 9 factors were included in a multivariate Cox proportional hazards model to adjust for the effects of covariates. Based on this model, tumor location, tumor size, *AP-2α* expression, T, N, and M stage remained independent prognostic factors (Table 2). The relative risk of death in patients with *AP-2α*-negative tumors was 1.512 times higher than that of patients with *AP-2α*-positive tumors (HR = 1.512, 95% CI = 1.127–2.029). Histological grade, radical resection, and chemotherapy, which were significant prognostic factors in the univariate analysis, did not show significant influence based on the multivariate analysis. We think that that the prognostic value of radical resection was overlapped by the M stage, and thus it did not show significant influence based on the multivariate analysis. Although patients in the adjuvant chemotherapy treatment group showed better overall survival rates based on the univariate analysis (P = 0.042), the lack of statistical significance in the multivariate analysis likely reflects a selection bias because adjuvant chemotherapy was more commonly administered to patients with advanced-stage disease.

**Table 2:** Univariate and multivariate survival analysis of clinic-pathologic variables in 481 cases of gastric carcinoma patients.

Variables	Univariate analysis			Multivariate analysis		
	HR	95%CI	P value	HR	95%CI	P value
Gender (male vs. female)	1.196	0.894–1.600	0.227			
Age (year), (≥60 vs. <60 )	1.293	0.979–1.707	0.070			
Location (distal/proximal/total)	0.725	0.558–0.940	0.015*	0.725	0.561–0.938	0.014*
Size (cm) (>5 vs. ≤5)	1.888	1.429–2.496	<0.001*	1.397	1.046–1.867	0.024*
Differentiation (G3/G2/G1)	1.376	1.060–1.786	0.017*	1.166	0.869–1.566	0.306
Radical resection (No vs. Yes)	4.603	3.373–6.282	<0.001*	1.312	0.659–2.610	0.439
AP-2α (low vs. high)	1.866	1.413–2.466	<0.001*	1.512	1.127–2.029	0.006*
T (T4b/T4a/T3/T2/T1)	1.784	1.504–2.117	<0.001*	1.469	1.210–1.784	<0.001*
N (N3/N2/N1/N0)	1.574	1.387–1.787	<0.001*	1.318	1.151–1.509	<0.001*
M (M1 vs. M0)	5.501	3.951–7.659	<0.001*	3.071	1.482–6.362	0.003*
Chemotherapy (No vs. Yes)	1.319	0.986–1.765	0.042*	0.972	0.714–1.322	0.856

*Statistically significant (*P*<0.05).

## Discussion

Tumor progression depends on factors that are intrinsic to the tumor cells, including, but not limited to, growth factors and their cognate receptors, extracellular matrix proteins, proteases, chemokines, and cellular adhesion molecules. The expression of these factors is influenced by the environment and the microenvironment, as well as by genetic and epigenetic factors. The transcription factor *AP-2α* has been shown to regulate many of the genes that are involved in normal cellular hemostasis [11], [15]. Therefore, the loss of *AP-2α* expression may result in dedifferentiation, proliferation, and eventually, metastasis or invasion [10], [12], [18]. Previously, *AP-2α* has been reported to possess tumor suppressive properties in breast cancer, ovarian cancer, prostate cancer and some other malignant tumors. However, to date, the prognostic significance of *AP-2α* in gastric cancer has not yet been evaluated.

In the present study, we investigated *AP-2α* mRNA and protein expression in primary gastric cancer specimens by RT-qPCR and western blotting detection, respectively. The transcriptional levels of *AP-2α* were determined with qRT-PCR and western blotting assays on 41 pairs of resected specimens (tumor tissue samples and matched adjacent non-tumor tissue samples) from gastric cancer patients. The results showed that the *AP-2α* mRNA and protein levels were significantly reduced in 30 (73%) and 28 (68%) tumor tissue samples, compared with levels in the adjacent non-tumor tissue samples (P = 0.009 and 0.012, respectively). These findings were consistent between RT-qPCR and western blotting detections. These observations support a previous hypothesis that *AP-2α* might be a tumor suppressor, and also suggest that *AP-2α* might play an important role in the tumorigenesis of gastric cancer.

Furthermore, in our study, which encompassed a relatively large series of gastric cancer patients (n = 481), we strengthened the hypothesis that *AP-2α* acts as a tumor suppressor in gastric cancer because low *AP-2α* expression was associated with poorly differentiated adenocarcinoma (G3), total gastric cancer, greater tumor progression (T4a/b and N3) and metastatic behavior (M1). Patients with low *AP-2α* expression were more likely to have stage III (44.5%) and IV (57.9%) disease than do patients with high *AP-2α* expression. These results are consistent with the findings of Gee et al. [19] and Pellikainen et al. [12], who described an association between high *AP-2* expression and high differentiation in breast cancer, including a low mitotic count and high histological grade (G1–G2). Similar findings have also been reported for other malignancies [10], [17], [18], [20]. Therefore, it seems that *AP-2α* maybe a potential tumor-suppressor gene involved in gastric cancer.

In the Kaplan–Meier survival analysis, patients with low *AP-2α* expression had a significantly shorter overall survival than those with high expressions. Univariate analyses showed that the decreased expression of *AP-2α* in gastric cancer tissues was significantly associated with overall survival rate. Multivariate analysis demonstrated that *AP-2α* expression, together with some traditional prognostic factors such as tumor location, tumor size, tumor depth, lymph node status, and metastasis status, were independent risk factors in the prognosis of gastric cancer patients. These results suggest that decreased *AP-2α* expression might help identify gastric cancer patients with a poor prognosis, and could therefore be a novel prognostic marker of gastric cancer patients.

In our study of gastric adenocarcinoma, we observed exclusively a cytoplasmic expression pattern of *AP-2α* proteins. However, both cytoplasmic and nuclear *AP-2α* expression have been previously described in several other malignancies [10], [12], [17], [20], [21]. In ovarian cancer, high cytoplasmic *AP-2α* expression is associated with a favorable prognosis; furthermore, in ovarian cancer, nuclear expression with low cytoplasmic expression is associated with an increased risk of death [17]. In breast cancer, combined cytoplasmic and nuclear *AP-2α* expression may provide important additional information on the prognosis and behavior of the disease [12]. In malignant melanomas, *AP-2* expression was shown exclusively nuclear expression [10]. In prostate carcinomas, the expression of *AP-2* was cytoplasmic in the majority of cases and nuclear expression of *AP-2* was present in 22% of the tumors [20]. However, both in colorectal and prostate carcinomas, cytoplasmic *AP-2* had been reported to have no prognostic value [20], [21]. As for the reason of above contradict results, we suppose that immunohistochemistry may only evaluate the end products of gene expression. Methodological factors, like antigen specificity, tissue processing and heterogeneity of different kind of malignancy may interfere with these results. Also, this phenomenon may partially due to modifications in the nuclear-pore complexes or in the activity of the transport receptors (karyopherines/importins/exportins) or changes of the *AP-2α* protein itself. In our results, reduced cytoplasmic expression of *AP-2α* predicted poor patients' outcome, suggesting that decrease of *AP-2α* transcription/translation or increased turnover rate are a more likely course than translocation in the case of gastric adenocarcinoma.

Although the possible relationship between decreasing the amounts of functional *AP-2α* and other cellular factors were not extensively investigated in the present study, the relatively large number of uniformly-treated patients strengthens the value of the current findings and significantly increases our knowledge of gastric adenocarcinoma patients.

In conclusion, the present study suggests that low *AP-2α* expression independently predicts worse overall survival in patients with gastric adenocarcinoma. However, the molecular mechanisms involved in the regulation of *AP-2α* expression in gastric cancer require further investigation. Future work in this field is necessary because greater understanding of *AP-2α* function in malignant transformation has the potential to improve prognostication in gastric cancer patients.

## Materials and Methods

### Ethics statement

The study was approved by the Ethics Committee of Sun Yat-sen University Cancer Center, and written informed consent was obtained from each subject.

### Patients

From January 2003 to December 2006, clinicopathological data from 481 gastric cancer patients who underwent surgical resection at Sun Yat-sen University Cancer Center were retrospectively analyzed. Patients who met the following eligibility criteria were included: (1) diagnosis of gastric adenocarcinoma identified by histopathological examination; (2) surgical history that included gastrectomy plus lymphadenectomy (limited or extended); (3) availability of complete follow-up data; (4) no preoperative treatment, such as chemotherapy and radiotherapy; (5) no history of familial malignancy or other synchronous malignancy (such as GIST, esophageal cancer, and colorectal cancer); (6) no recurrent gastric cancer and remnant gastric cancer; and (7) no death in the perioperative period. Tumor resection and D2 lymphadenectomy were performed by experienced surgeons, and the surgical procedures, which followed the Japanese Gastric Cancer Association (JGCA) guidelines [22], were similar in all patients who underwent radical resections.

### Tissue specimens

Fresh gastric cancer and adjacent non-tumor tissue samples (n = 41) were obtained from 41 gastric cancer patients who underwent surgical resection at the Sun Yat-sen University Cancer Center between 2005 and 2006. These 41 patients included 27 males and 14 females, with a median age of 50 years (range, 21–75 years). After surgical resection, the fresh tissue samples were immediately immersed in RNAlater (Ambion Inc., USA) and stored at 4°C overnight to allow thorough penetration of the tissues; the samples were then frozen at −80°C until RNA extraction. Both the tumor tissue and the adjacent non-tumor tissue, which was located more than 2 cm away from the gastric cancer, were sampled and then verified by pathological examination.

Paraffin-embedded samples were obtained from 481 gastric cancer patients who underwent surgical resection at the Sun Yat-sen University Cancer Center between 2003 and 2006. These patients included 326 male and 155 female patients, with a median age of 58 years (range, 17–85 years). Each tumor sample was assigned a histological grade based on the World Health Organization (WHO) classification criteria. All patients were staged using the 7th edition of the International Union Against Cancer (UICC) Tumor-Node-Metastasis (TNM) staging system.

### Extraction of total RNA and RT-qPCR

Total RNA was extracted using TRIzol solution (Invitrogen, USA) according to the manufacturer's protocol. RNAse-free DNAase I was used to eliminate DNA contamination. Total RNA concentration and quantity were assessed by absorbency at 260 nm using a NANO DROP spectrophotometer (ND-1000, Thermo Scientific, USA) and the purity of the samples was estimated by the OD ratios (A260/A280, ranging within 1.8–2.2), visualized on an agarose gel to check quality. Reverse transcription (RT) was performed in a 25 µl reaction volume with 2 µg total RNA treated with 0.5 µg of Oligo(dt), 200 U M-MLV reverse transcriptase, 25 U RNase inhibitor and 2.5 mM dNTP to synthesize first-strand cDNA (Promega, USA), according to the manufacturer's recommendations. The reaction system was incubated at 70°C for 5 minutes (primer annealing), 42°C for 1 hour (synthesis) and resulting cDNA was stored at −20°C. The resulting cDNA was subjected to RT-qPCR for the evaluation of the relative expression levels of GAPDH (as an internal control) and *AP-2α*. The sequences of the sense and antisense primers were as follows: 5′-AGGGCGAAGTCTAAAAATGGAG- 3′ (F) and 5′- TAGTGATGTGAGCAGGGTAACG-3′(R) for *AP-2α*; 5′- CTCCTCCTGTTCGACAGTCAGC-3′(F) and 5′- CCCAATACGACCAAATCCGTT -3′(R) for GAPDH, and the corresponding PCR products were 114 bp and 113 bp, respectively. Gene-specific amplification was performed using an Applied Biosystems (ABI 7900HT) RT-qPCR machine that measured the binding of SYBR Green I to double-stranded DNA. Each sample was tested with a no template control (NTC) for each pair of oligonucleotide primers to control contamination or primer-dimers, and each experiment was repeated at least twice using cDNA samples from separate reverse transcription reactions.

The reactions were performed in a total volume of 15 µl that contained the following: 0.5 µl cDNA that was synthesized as described above, 7.5 µl of 2×SYBR Green master mix (Invitrogen, USA), and 200 nM of each pair of oligonucleotide primers. The amplification was performed as follows: an initial step at 95°C for 10 min, followed by 45 cycles of 95°C for 30 sec and 60°C for 60 sec. Regression curves were calculated for each sample, and the amplicated sample's relative quantity was calculated from the threshold cycles using the instrument's software (SDS 2.0). The RT-qPCR amplicons were analyzed with gel electrophoresis to confirm the specificity of the generated products. Relative expression levels of the target genes were normalized to the geometric mean of the internal control gene, GAPDH. The generated data were averaged and expressed in relative units of normalized expression. Data were analyzed using the comparative threshold cycle (2−ΔΔCT) method.

### Western blotting analysis

The frozen tissue samples from patients with gastric cancer including the tumor and non-tumor tissue, were homogenized in RIPA lysis buffer, and the lysates were cleared by centrifugation (12,000 rpm) at 4°C for 15 min. Approximately 40-µg protein samples were run on a 12% SDS-PAGE gel and were transferred to PVDF membranes. After blocking non-specific binding sites for 60 min with 5% non-fat milk, the membranes were incubated overnight at 4°C with a primary polyclonal antibody against *AP-2α* at a 1∶2000 dilution). The membranes were then washed three times with TBST for 10 min each and probed with HRP-conjugated secondary antibody (at a 1∶2000 dilution) for 60 min at room temperature. The membranes were then washed three times with TBST and developed with an enhanced chemiluminescence system (ECL, Pierce).

### Immunohistochemistry

Formalin-fixed, paraffin-embedded samples were sectioned at a thickness of 2 µm, and the sections were deparaffinized and rehydrated using graded ethanols. For antigen retrieval, the slides were boiled in EDTA (1 mM; pH 8.0) for 15 min in a microwave oven. Endogenous peroxidase activity was blocked with 0.3% hydrogen peroxide solution for 10 min at room temperature. After rinsing with PBS, the slides were incubated overnight at 4°C with a 1∶500 dilution of rabbit anti-*AP-2α* monoclonal antibody (Santa Cruz, USA). After three washes in PBS, the sections were incubated with biotinylated secondary antibody (Zhongshan Golden Bridge Biotech., Beijing, China) for 30 min at room temperature. Finally, the visualization signal was developed with 3,3′-diaminobenzidine tetrahydrochloride (DAB), and all of the slides were counterstained with hematoxylin.

### Semi-quantitative methods

The specimens were analyzed by three observers (W. W., L. L., and Y.Z.) who were blinded to the patients' clinical outcomes. Discrepancies between the observers were found in less than 10% of the examined slides, and consensus was reached after further review. The total *AP-2α* immunostaining score was calculated as the sum of the percent positivity (the percentage of the positively stained tumor cells) and the staining intensity. The percent positivity was scored as “0” (<5%, negative), “1” (5–25%, sporadic), “2” (25–50%, focal), or “3” (>50%, diffuse). The staining intensity was scored as “0” (no staining), “1” (weakly stained; visible at high magnification), “2” (moderately stained; visible at low magnification), or “3” (strongly stained; strikingly positive at low magnification). The total *AP-2α* immunostaining score was calculated with the value of percent positivity score×staining intensity score, which ranged from 0 to 9. We defined the high *AP-2α* expression level as a total score ≥4, and low *AP-2α* expression as a total score <4.

### Follow-Up

Postoperative follow-up occurred at our outpatient department and included clinical and laboratory examinations every 3 months for the first 2 years, every 6 months during the third to fifth years, annually for an additional 5 years or until patient death, whichever occurred first. Overall survival, which was defined as the time from the operation to the patient's death or the last follow-up, was used as a measure of prognosis.

### Statistical analysis

A paired-samples t-test was used to compare the *AP-2α* mRNA levels in the tumor tissue samples and the adjacent non-tumor tissue samples. The χ2 test for proportion and Pearson's correlation coefficients were used to analyze the relationship between *AP-2α* expression and various clinicopathological characteristics. Overall survival curves were calculated with the Kaplan-Meier method and were analyzed with the log-rank test. Cox proportional-hazard analysis was used for univariate and multivariate analysis to explore the effect of clinicopathological variables and *AP-2α* expression on survival. A two-sided P-value<0.05 was considered to be statistically significant. All statistical analyses were performed with SPSS software (version 16.0; SPSS Inc., Chicago, IL, USA).
